# Working Together and Being Physically Active Are Not Enough to Advise Uniformly and Adequately Low Back Pain Patients: A Cross-Sectional Study

**DOI:** 10.1155/2018/4128913

**Published:** 2018-06-26

**Authors:** C. Praz, J. Ducki, M. L. Connaissa, P. Terrier, P. Vuistiner, B. Léger, F. Luthi

**Affiliations:** ^1^Institute for Research in Rehabilitation, Clinique Romande de Réadaptation Suvacare, Avenue Grand-Champsec 90, 1950 Sion, Switzerland; ^2^Institute of Sports Sciences, University of Lausanne, Géopolis, Campus Dorigny, 1015 Lausanne, Switzerland; ^3^Department for Musculoskeletal Rehabilitation, Clinique Romande de Réadaptation Suvacare, Avenue Grand-Champsec 90, 1950 Sion, Switzerland; ^4^Department of Physical Medicine and Rehabilitation, Orthopaedic Hospital, Lausanne University Hospital, Rue du Bugnon 21, 1011 Lausanne, Switzerland

## Abstract

The profession of the health-care providers (HCPs) influences their recommendations to the patients. Conversely, interdisciplinarity seeks to challenge such differences, so that the patient receives one single and consistent therapeutic message. Some studies also suggest associations between HCPs life habits and recommendations. Our hypotheses were (1) that despite interdisciplinary work, the profession remains a predictor of recommendations and (2) that HCPs who are more physically active recommend more activity. Three clinical vignettes were presented to a group of experts of low back pain (LBP) (guidelines), and 20 physicians, 22 physiotherapists, and 23 nurses to assess how they evaluate the symptoms and pathologies of LBP patients and how much work and physical activity they recommend. Physical activity was assessed with accelerometers and questionnaires. Some interprofessional differences remained present within an interdisciplinary team. The nurses were more restrictive and further away from the guidelines. The physicians were the most in line with them. The physiotherapists recommend as much physical activity, but less work activity than the physicians. The level of physical activity of the HCPs is not associated with their recommendations. To ensure a clear and unique message, educational actions may be undertaken to promote the biopsychosocial model and clarify the guidelines.

## 1. Introduction

Interdisciplinarity has become a key concept in health care. It allows a comprehensive approach to develop consensual guidelines and recommendations [1]. According to Mitchell et al. [2] five key principles underpin effective team-based care: shared goals, clear roles, mutual trust, effective communication, and measurable processes and outcomes. To be efficient, interdisciplinarity should ensure that the patient gets one single and consistent message through uniform recommendations from all the health-care providers (HCPs) within a team [2, 3]. However, it is still unclear to what extent individual HCPs' characteristics, such as professional background or personal believes, may prejudice the coherence of recommendations. Notably, no one has examined if the interdisciplinary work decreases the interprofessional differences of recommendations.

What is already known is that individual beliefs and life habits may affect attitude and recommendations of HCPs notably regarding low back pain (LBP) [4–10]. Likewise, two studies showed that chiropractors endorse a more biomedical approach than physiotherapists and osteopaths [4, 11]. Another study demonstrated that physiotherapists, nurses, and physicians have different beliefs about movement and LBP [12]. Concerning life habits, HCPs with a higher level of physical activity (PA) may recommend more activity to their patients [13–17]. Lobelo and deu Qevedo also highlighted the role of the HCPs as models for patients regarding PA [18]. Despite this abundance of evidence of a large influence of individual traits and professional background on recommendations, it is still unclear whether interdisciplinary work tends to equalize recommendations among team members.

Interdisciplinarity is important in LBP care and rehabilitation [19]. LBP is indeed a very highly prevalent condition [20–24]. However, causes, prognosis, and course are often undefined, leading to important variations in clinical practice [25]. Two main treatment orientations are defined: the biomedical orientation that considers that pain is always caused by a lesion and that it may justify disability and the avoidance of activities and the today recommended biopsychosocial model that takes into account biological, psychological, and social factors and promotes early return to activity and work [5, 7, 26–29]. Despite the guidelines, it was reported that many HCPs are still too restrictive and believe that LBP requires some avoidance of activities and to remain off work [5]. Simmonds et al. showed that only 12% of the physiotherapists identify correctly the guidelines, and most of them do not agree with them regarding return to work or activity [30]. To summarize, however, interdisciplinarity is particularly important in LBP care, a high risk of divergence exist among HCPs' recommendations, which deserves further investigation.

Our main objective was to evaluate the coherence of PA recommendations to patients within an interdisciplinary team of physicians, physiotherapists, and nurses. We postulated that if the team worked coordinately, the members of the team should give similar recommendations regardless of the professional background. Conversely, in case of a strong professional group effect, the recommendations could differ despite the interdisciplinary work.

Our secondary objective was to test whether recommendations depended on PA level and personal beliefs. Specifically, the hypotheses were (1) more physically active HCPs would recommend more PA and (2) HCPs that perceived themselves as more active would recommend more PA.

## 2. Materials and Methods

### 2.1. Setting

The study included HCPs of the Clinique Romande de Réadaptation, a teaching rehabilitation centre in the French-speaking part of Switzerland. Patients with chronic LBP represent a large proportion of the musculoskeletal patients' population (about 25% of the patients, 250 cases/year). The aim of the administrated therapeutic program (4 to 5 weeks with at least 3 to 4 h of daily therapies excluding weekends) is to manage pain and to improve function, activity, and participation, including return to work (usual or adapted), using a multidisciplinary biopsychosocial approach according to the recommended practice for patients with chronic pain [31].

The rehabilitation centre strongly encourages interdisciplinary work and makes efforts to implement shared goal, clear role for each HCP, mutual trust, effective communication, and measurable processes and outcomes, as advanced by Mitchell et al. [2]. Every week, the HCPs of the different professional groups meet to discuss the cases and plan therapeutic goals according to the ICF framework (taking into account the personal and environmental factors, which could influence the recovery). Additionally, internal trainings common for all the professional groups are organized, including training about the biopsychosocial model and about fear-avoidance beliefs. These trainings take place at least twice a year, and all the HCPs should be familiar with the fear-avoidance and biopsychosocial models [32, 33].

The therapeutic team is mainly composed of physicians, nurses, and physiotherapists. Occupational therapists, education and vocational guidance professionals, and psychologists are also members of the team, but they are far less numerous and were therefore excluded from this study for statistical reasons. During the rehabilitation program, the physicians are responsible for the diagnosis, the prescription of medication and treatment, the coordination of the rehabilitation program, and the follow-up of the patients. The physiotherapists assume the therapeutic training, the functional tests, the reconditioning (strengthening and aerobic exercises), stretching, and manual therapy; they are specialized in the rehabilitation of the musculoskeletal system. The nurses are responsible for the assessment of the biopsychosocial complexity, for the training for self-administrated medication, for counseling for the management of activities of daily living, and upon request for the psychological support and assistance. Moreover, the nurses conduct patients' interviews at the beginning of the rehabilitation program, according to the INTERMED tool, to detect biopsychosocial complexity [34–36].

### 2.2. Participants

We invited 97 HCPs working in the centre as physicians, physiotherapists, or nurses to participate in the study. Information about the study was given during the weekly teaching team meeting of each professional group. The HCPs who were absent on the day of the meeting were excluded from the study. The participants were informed of the procedures involved in the study and gave written informed consent.

### 2.3. Design

We conducted a cross-sectional survey in January 2015. The study consisted of clinical vignettes and questionnaires that the HCPs have to fulfil during questionnaire sessions and objective measurements of the PA with accelerometers. The accelerometers were distributed to HCPs between November 2014 and February 2015, according to their availability. The local Research Ethics Committee granted the approval to undertake the study (CCVEM 041/14).

### 2.4. Primary Outcome: Severity and Recommendations

To assess the effects of the professional discipline and the level of PA performed by the HCPs on their therapeutic advices, the HCPs were asked to evaluate three hypothetical medical cases and to give their recommendations for it.

Three clinical vignettes as described by Rainville et al. [28] were used to assess the beliefs and attitudes of HCPs regarding patients with chronic LBP. The vignettes describe cases with working situation, symptoms, relevant physical findings, results of examinations, and previous treatments. All vignettes suggested LBP with a nonspecific cause. For each vignette, the HCPs were asked to answer four questions on a one to five Likert scale about (1) the severity of the symptoms (from “very mild” to “extremely severe”), (2) the severity of pathology (same scale), (3) the recommendations for the level of activity (from “no activity limitations” to “limit all physical activities”) and (4) the recommendations for the work activity (from “full time” to “remain out of work”). Higher scores indicate recommendations that are more restrictive and symptom and pathology perceptions that are more severe. The three vignettes and the corresponding questions were translated into French by a professional translator. Then members of the research team who are fluent in French and English assessed and verified the translation. The outcome of the HCPs was compared with the evaluation and the recommendations of a group of respected experts in the field. This group consisted of twelve specialists of LBP: seven physicians and five physiotherapists from three different medical centres in Switzerland (teaching rehabilitation centres and university hospitals). To be considered as an expert, they had to spend more than 80% of their working time with patients with LBP for at least 10 years and to be referring physicians or physiotherapists for LBP in their institution. The experts answered the four questions about the three vignettes and their average opinion for each item is considered as a guideline to get the best treatment for patients with LBP.

### 2.5. Variables with Potential Effect on the Recommendations

Six different self-administrated questionnaires were completed by the HCPs to assess some variables with a potential effect on the evaluation and recommendations of the clinical vignettes.

#### 2.5.1. Sociodemographic

Demographic data were collected which included age, gender, working discipline, working experience, training in LBP, and LBP history (present or previous).

#### 2.5.2. Activity Pattern

The Pattern of Activity Measure (POAM-P) is usually administered to assess the activity pattern in chronic pain patients [37]. POAM-P allows the identification of three different activity patterns: “avoidance,” “pacing,” and “overdoing.” We used the validated French version of the POAM-P [38]. Since this questionnaire has been designed for chronic pain patients, we modified the instructions asking the HCPs to imagine themselves suffering of chronic LBP. The questionnaire is composed of 30 items, 10 items for each pattern. For each item, the participants were asked to describe the extent to which the selected item describes how they would perform their activity. A Likert scale was used, ranging from zero “not at all” to four “all the time.” For each pattern of behaviour, a score from 0 to 40 was obtained. A higher score indicated that the participant would be more likely to use the considered activity pattern.

#### 2.5.3. Pain-Related Fear of Movement

The Tampa Scale for Kinesiophobia (TSK) was originally designed to evaluate the fear of movement and/or (re)injury in patients with LBP [39]. The TSK is composed of 17 items. Each item is rated on a four points Likert scale ranging from one, “strongly disagree,” to four, “strongly agree.” Overall score lies between 17 and 68 [40, 41]. A higher score indicates a higher level of kinesiophobia. Houben et al. adapted the questionnaire for HCPs by changing the instructions to determine to what extent HCPs believe that movement and PA are harmful for their LBP patients [4].

#### 2.5.4. Treatment Orientation

The Health Care Providers' Pain and Impairment Relationship Scale (HC-PAIRS) is a questionnaire that estimates the attitudes and beliefs of HCPs about functional expectations for chronic LBP patients [42]. The HC-PAIRS consists of 15 statements about the functional expectations for patients with chronic back pain. Each statement is evaluated on a seven point Likert scale, from one, “totally disagree,” to seven, “totally agree,” on which the HCPs marked their level of agreement with that statement. By summing the results of the 15 items, we got a score from 15 to 105. Higher scores suggested that the HCP has a more biomedical approach of LBP and is more likely to agree with the fact that back pain necessitates the avoidance of activities and justifies disability. In contrast, lower scores are linked with a more biopsychosocial approach. This questionnaire was send afterward by email.

#### 2.5.5. Anxiety and Depression Symptoms

The HCPs completed the validated French version of the Hospital Anxiety and Depression Scale (HADS) [43, 44] to measure their tendency to be anxious and depressed. This self-administrated questionnaire was originally designed for hospitalized patients but has been also validated for the general population [45]. The HADS is composed from two subscales: one for anxiety symptoms and one for depression symptoms. Each subscale includes seven items and each item is rated on a zero to three Likert scale. Therefore, the subscores for both anxiety and depression are ranging from 0 to 21. Higher scores indicate more anxious/depressive symptoms. There is evidence of a relationship between the HADS scores and the patient's pain chronification [46].

#### 2.5.6. Intolerance of Uncertainty

The Intolerance of Uncertainty Scale (IUS) is a 27-item questionnaire used to assess the belief that uncertainty is unacceptable and leads to frustration, stress, and the inability to take action [47]. Each item is evaluated on a one to five scale from 1, “not at all characteristic of me,” to 5, “entirely characteristic of me.” It results with scores between 27 and 135. Higher scores represent greater intolerance of uncertainty (IU). Simmonds et al. has demonstrated a positive association between IU and biomedical orientation [30].

### 2.6. Perceived and Objective Physical Activity Level

We used questionnaires and accelerometers to evaluate the PA level.

#### 2.6.1. Self-Reported Physical Activity

To assess the HCPs' habitual PA, the Baecke habitual physical activity questionnaire [48] was completed. It is a brief questionnaire of 16 items, translated and validated in French [49]. It consists of three sections assessing, respectively, the PA at work (work index), at sport (sport index), and during leisure time, excluding sport (leisure time index). Every domain results in a separate subscore, and the overall score is calculated by averaging the three subscores. The results are given in metabolic equivalent of task (METs) [50]. This measure is subjective and represents the perceived PA of the participant.

#### 2.6.2. Objective Physical Activity

The participants' objective PA level was assessed during a normal working week, from Tuesday to the following Sunday (six consecutive days). The PA was measured with a validated three-axis microelectromechanical system (MEMS) accelerometer, the ActiGraph wGT3X-BT (ActiGraph, Pensacola, USA). The ActigGraph recorded body accelerations continuously (sampling rate: 50 Hz). The sensor is a small and light box (19 g) attached on an elastic belt, and that should be worn on the left hip. Participants were instructed to wear the device during waking hours, from awakening to bedtime to measure their habitual PA. The accelerometer detected when and how much the HCPs were walking. The number of steps per day was calculated and considered as a marker of the HCP's PA [51].

For the analysis, the days with less than 12 h of acceleration data were discarded. We also discarded rest days, according to the working schedule of each participant. The Matlab software (Mathworks, Natick, MA, USA) was used for data analysis. An algorithm specifically recognized walking through signal amplitude and frequency pattern and computed the average number of steps per day performed by the subjects [52]. The number of steps is deduced from the duration of each walking bouts and the cadence (i.e., average number of steps per second). Total daily number of steps is the sum across all the walking bouts. The average number of daily steps per day over the week is finally computed.

### 2.7. Potential Bias

Different measures were taken to avoid or evaluate the bias that could interfere. Firstly, all the HCPs of the same professional group answered the questionnaires on the same day during the same weekly meeting to avoid contamination of the data by discussions and mutual influence. Secondly, we assessed social desirability that is the tendency of the respondents to answer questions in a manner that will be viewed favorably by others. Social desirability was used to evaluate to what extent it was possible to rely on the answers as a reflection of the real behaviour of the HCPs. The Marlowe–Crowne Social Desirability Scale (MC-SDS) measures the inclination to respond in a socially desirable way [53]. The MC-SDS questionnaire consisted of 33 statements to which participants were asked to answer “true” (one point) or “false” (zero point). It results in a score from 1 to 33. Scores ≥ 20 indicate a high social desirability. This questionnaire allows assessing the validity of the scores calculated for all the other self-administered questionnaires.

### 2.8. Data Analysis

Data were analysed using Stata 13.1 (StataCorp, College Station, TX, USA). For each variable, the means (M) and standard deviations (SD) were calculated for the three professional groups and for all the HCPs. Analysis of variance (ANOVA) followed by post hoc Scheffe multiple-comparison tests (for continuous data) and chi-squared tests (for categorical data) was performed to measure the associations between the professional groups and the different variables. In addition, *η* ^2^ was used as an effect size measure, as it can be interpreted as the proportion of variance in the dependent variable that is attributable to the professional groups. The significance level was set at *p* < 0.05.

The mean responses of the experts were considered as the guideline. For each HCP, the discrepancies between their answers and the guideline were expressed with the bias (raw difference between the answer of the HCP and the guideline) and the intraclass correlation coefficient (ICC) calculated with Shrout and Fleiss form 2.1 [54]. Bias was calculated for the average of the three questions (one for each vignette) about severity of symptoms (bias_sev-sympt_), for the average of the three questions about severity of pathology (bias_sev-patho_), for the average of the three questions about recommendations for daily PA (bias_rec-act_), and for the average of the three questions about recommendations for work (bias_rec-work_). ICC was calculated for the average of the six questions about severity (two for each vignette) (ICC_sev_) and for the average of the six questions about the recommendations (ICC_rec_). We did not calculate the ICC for each of the four subscales separately because the number of items was not sufficient to get statistically significant ICC values.

For the PA (objective and perceived), the Pearson correlations between the subscores on Baecke (perceived) and the number of steps per day (objective) and the evaluation of severity and the recommendations were also determined independently of the professional groups.

## 3. Results

### 3.1. Response Rate

Ninety-seven HCPs were invited to participate during their weekly team meetings. Seventy-two HCPs gave written informed consent, which results in an overall inclusion rate of 74%. Seven HCPs were excluded due to incomplete data. Finally, 65 HCPs were considered for the analysis: 20 physicians, 22 physiotherapists, and 23 nurses. The HC-PAIRS, which was sent afterward, was answered by 56 HCPs only (14 physicians, 19 physiotherapists, and 22 nurses).

### 3.2. Subjects' Characteristics

The main anthropometric and professional characteristics of the subjects are summarised in Table 1. The majority of the physiotherapists and nurses were women (68% and 70%), while only 40% of the physicians were women. However, this difference was not significant (*p*=0.09). The physicians tend to be older than the nurses (significant Scheffé's post hoc comparison). A large majority of the physicians have a special training external to the medical centre for LBP and/or pain (85% for LBP and 90% for pain). It is less the case for physiotherapists (73% and 45%) and it is much less common for nurses (22% and 35% resp.) (significant difference between the professions (*p* < 0.01)).

Body mass index (BMI) was not significantly different among the professional disciplines. No significant interprofessional difference was found for the number of years of working experience and the prior and current LBP experience.

### 3.3. Responses of the Experts

The group of experts answered the four questions about the three clinical vignettes.  Table 2 presents the average scores of the 12 experts.

### 3.4. Primary Outcome: Comparison of the Evaluation and Recommendations between Professional Groups and Discrepancies with the Experts

Overall, the total variance explained by professions was moderate (20%–30%, *η* ^2^, Table 3). The highest between-group difference was found for work recommendation (*η* ^2^ = 0.3).

The physicians evaluated the overall severity as less severe than physiotherapists and nurses. A more detailed analysis showed that the difference between the physiotherapists and the physicians was not significant for the severity of symptoms but was significant for the severity of the pathology, while the differences between the nurses and the physicians were significant for the severity of the symptoms and for the severity of the pathology.

In average, the nurses recommended less activity (daily and work activity) than the physicians and the physiotherapists. Concerning PA recommendations, nurses recommended significantly less daily PA than the physicians and the physiotherapists. Concerning work recommendations, the physicians recommended more work than the nurses and the physiotherapists.

The answers of the HCPs were compared with the experts. The bias and the ICC are presented in Table 3. Differences between professions were observed on bias_sev-symp_ (physicians significantly different from nurses), on bias_seve-patho_ (physicians significantly different from physiotherapists and nurses), on bias_rec-act_ (nurses significantly different from physicians and physiotherapists), and on bias_rec-work_ (physicians significantly different from physiotherapists and from nurses). ICC_rec_ tended to be different (*p*=0.05) across the professional groups, while ICC_sev_ was not different (*p*=0.24).

### 3.5. Variables Potentially Associated with the Recommendations

The main scores of the questionnaires are summarised in Table 4.

There were significant differences across the three professional groups for the POAM-P (avoidance score), the TSK, the HC-PAIRS, the HADS (both anxiety and depression), and the IUS.

Nurses have a greater tendency to think that they would avoid activity if they suffered from LBP. For the pacing and overdoing subscores, no significant difference was found between the three groups.

The TSK score was also significantly different between the professions (*p* ≤ 0.01). The belief that movement and PA are harmful for LBP patients was more developed by nurses. They were significantly more afraid of movement and (re)injury for their patients than physicians and physiotherapists.

The HC-PAIRS score indicated significant differences between the professions in the treatment orientation (*p* ≤ 0.01). The nurses had the most biomedical approach (65 (9)), whereas the physicians the less biomedical approach (52 (7)); the physiotherapists were in between. The HC-PAIRS scores of nurses were significantly higher than those of the physicians and the physiotherapists.

HADS scores were significantly different between the professional groups (*p*=0.02 for both anxiety and depression). Nurses showed the most anxious and depressive symptoms while the opposite was observed for the physiotherapists.

For the IU, the mean score was 47 (12) with a significant difference between the professional groups (*p*=0.03). The nurses tended to be the most intolerant to uncertainty (51 (14)), significantly more than physicians. The scores of the physiotherapists were once again in between.

### 3.6. Secondary Outcomes: Perceived and Objective Physical Activity Level

#### 3.6.1. Self-Reported Physical Activity

The HCPs' mean activity level assessed with the Baecke questionnaire (Table 5) was 3.1 ± 0.4 METs, which is considered as moderate (from 3 to 4.9 METs [50], physician's subscore for the work index was the only one classified as low (2.2 (0.4)). Physician's score was significantly lower than the score of the physiotherapists and the nurses. Nurses had the lowest sport and leisure time indexes when compared to physiotherapists and physicians, respectively.

Independently of the professional group, the work index was significantly correlated with the global evaluation of severity of symptoms and pathology (*r*=0.34, *p* < 0.01) and with the recommendations for work and daily activity (*r*=0.47, *p* < 0.01). The HCPs who considered themselves as less active at work are those who considered the severity as lower and who recommended more activity.

#### 3.6.2. Objective Physical Activity

Table 5 compares the number of steps per working day of the different professional groups. According to Tudor-Locke et al., 10,000 steps per day is a reasonable target for a healthy lifestyle for adults [51]. On average, the HCPs did not reach the target (7,812 (2,699)). The difference between the groups was significant (*p* < 0.01). The physiotherapists were the most active during working days. The physicians walked significantly less than physiotherapists and nurses.

Independently of the professional group, no significant relationship was found between the number of steps per day and the evaluation and recommendations for the clinical vignettes.

### 3.7. Social Desirability

The mean score on MC-SDS was 19.2 ± 4.3 with no significant difference between the professional groups (Table 4). The cut-off score that define a high social desirability is set at 20. 32 subjects (49%) reached scores over the cut-off.

## 4. Discussion

### 4.1. Key Results (Primary Outcome)

We aimed at investigating the influence of the profession on the recommendations given to the LBP patients within an interdisciplinary team. Our results suggest that despite interdisciplinary team working, each profession keeps its own beliefs and attitudes. Physicians thus considered symptoms and pathology as less severe than the other professions and were more in line with guidelines. Conversely, nurses diverged substantially from guidelines. In fact, they recommended more limitation of work and PA. Physiotherapists ranged generally between the two other groups; their recommendations for PA were similar to physicians' recommendations, whereas their recommendations for work were closer to those of the nurses.

### 4.2. Interpretations of the Interprofessional Difference

We observed a strong influence of professional background on recommendations and beliefs. HCPs that were less in accordance with the guidelines and gave more restrictive PA and work recommendations (i.e., nurses) had the following characteristics: (1) they had a more biomedical approach; (2) they were more likely to behave with an “avoidance” pattern; (3) they were more kinesiophobic and (4) intolerant to uncertainty, and (5) they had more anxious and depressive symptoms.

Generally, our results are consistent with previous studies. Houben and colleagues have noted an association between the TSK score, the HC-PAIRS score, and the recommendations for the clinical vignettes [4, 10]. Likewise, an association between the subscore “avoidance” (POAM-P) and the TSK score has been observed, but only for patients so far [37]. Simmonds et al. have shown that the greatest IU is associated with a more biomedical approach and more developed anxious symptoms [30, 55, 56]. All of these factors seem to influence each other and to have an effect on the recommendations.

The nurses diverged substantially from the experts (Table 3). They tended to recommend less activity than other HCPs. Nurses' beliefs (Table 4) confirmed a trend toward more fear of movement and a more biomedical approach. Paradoxically, in our institution, nurses follow regular training on the biopsychosocial model and play an important role to determine patients' biopsychosocial complexity. Differences between nurses' recommendations and the guidelines are especially concerning because their role is of prime importance in health care [57]. Unfortunately, their recommendations and believes were often neglected so far. The lack of basic training and knowledge may be a reason for the discrepancy with the guidelines. Dermody et al. showed that young nurses perceived that they lack knowledge and training to safely mobilize hospitalized patients [58]. In our sample, the nurses were the HCPs, who have less formalized professional training on LBP and chronic pain in general (Table 1). This may be related to our clinic or more likely to the Swiss health care system, which has only recently developed such special trainings available for nurses (e.g., certificate of advanced studies). Accordingly, nurses should benefit from more training about chronic pain during their basic training and afterward.

The physiotherapists were often between nurses and physicians regarding attitudes and beliefs. Concerning recommendations, the relevant differences with the physicians were for the severity of pathology, and the recommendations for work, physiotherapists were more restrictive than physicians. This may be because the physicians are especially trained to diagnose pathologies and to certificate medical leave [59]. Conversely, the physiotherapists' training on LBP and chronic pain is often more focused on biomedical approach (e.g., manual therapy) than on exercise therapy and on the biopsychosocial approach although the biopsychosocial approach is also part of their training and profession. Moreover Simmonds et al. demonstrated that only 12% of the physiotherapists identified correctly the guidelines, while Mikhail et al. showed that even when they were aware of recommendations, they did not necessarily implement them [30, 60].

The physicians were more in line with the experts. Thus, they evaluated the symptoms and the pathology as less severe than the other professions and recommended a higher level of activity and work. A possible explanation could be that physicians are generally well trained in pain and LBP management (Table 1). In addition, physicians exhibit greater specialization in the return to work process. Physicians' educational background in rehabilitation medicine favors a biopsychosocial orientation [61]. The physicians spend also less time with the patients (about once a week) than physiotherapists (several times a week) and nurses (extended period every day). Thus, the physiotherapists and especially the nurses may consider they have a supportive role to play, which may lead them to be less pushy and rigorous.

These results highlight the necessity to improve the educational background of HCPs that play a key role with the patients.

### 4.3. Additional Results (Secondary Outcome): Perceived and Objective Physical Activity Level

The hypothesis that more active HCPs would recommend more PA for their patients is not supported by the results. On the contrary, we observed that the less active HCPs (i.e., physicians) recommend more PA. Physician's worktime is mostly dedicated to patients' examination and administrative work, which is not conductive for a high PA level. Likewise, physiotherapists' engagement in physical therapies makes them the most active professional group, but they do not recommend the most PA. So it is probably an effect of the profession rather than a real association between low PA level and more activity is recommended. Furthermore, Baecke's work index was negatively correlated with the recommendations. In other words, concerning PA recommendations, professional background seems dominate over personal PA practice. Previous studies suggested a possible effect of the perceived PA on the recommendations [18]. By using both subjective and objective PA measures, we cannot confirm that physically active HCPs are more likely to provide PA counseling to their patients and can indeed be PA role models.

### 4.4. Strengths of the Study

The originality of our study resides in the inclusion of a group of experts to establish valid answers according to the guidelines. The main advantage was that the experts gave their opinion about practical cases that can be directly compared with those of the HCPs.

The assessment of both perceived and objective PA is another strength of the study; in this way, the different facets of PA that may affect the recommendations were measured.

Through the MC-SDS questionnaire, we take into account social desirability. The MC-SDS scores are moderate. Therefore, the other questionnaires probably reflect the real beliefs and attitudes of the HCPs. Importantly, the MC-SDS scores were not significantly different among the three professional groups. Between-group comparisons were thus not biased.

### 4.5. Limitations of the Study

A limitation of our study is that we did not observe the actual HCP's professional practice and behaviour. We only relied on their subjective opinion facing hypothetical cases (vignettes) and situations (questionnaires).

The limited sample size is also a limitation. As we want to compare all HCP working within the same team, it is rather difficult to include larger sample size.

Another limitation may come from the selection of the experts. The fact that the experts were either physicians or physiotherapists may limit the interpretation of the differences observed between the nurses and the others professional groups.

The use of the number of steps to assess PA is also limitation because it neglects sport activities, in particular cycling, strength training, or swimming. Additionally, it would be interesting to assess the PA during days off to have a more global view of the PA with more leisure time.

Finally, our study was conducted in a teaching centre, whose characteristics might not be comparable to other settings. Further studies are necessary to verify the external validity of our findings.

## 5. Conclusion and Perspectives

Our main finding is that, despite working together and sharing similar regular teaching meetings, HCPs deliver recommendations that substantially differ according to their professions. With that in mind, efforts should be made to harmonize the beliefs, attitudes, and recommendations. These efforts should be especially directed to the nurses and physiotherapists whose approach of pain and treatments is more biomedical and further from the guidelines. In our opinion, a better anchoring of the biopsychosocial model must be obtained through a focused training of the new HCPs and dedicated information to medical centres. The importance of training was highlighted by Kennedy et al. who showed that the beliefs of students in medicine and physiotherapy are closer to the guideline at the end of their studies compared to the beginning [12]. Furthermore, even a short training about the biopsychosocial model may already change the treatment orientation of physiotherapists [6, 8, 12, 62]. In our centre, HCPs follows regular theoretical trainings about the biopsychosocial and fear-avoidance models. It may be more profitable to integrate practical training, with discussions of real cases or vignettes. These trainings should also preferably mix the professions to encourage the HCPs to discuss the cases and the recommendations together. Additionally, it would be interesting thereafter to test whether such trainings are translated into the real-life practice and may improve patients' outcomes.

## Figures and Tables

**Table 1:** Characteristics of the professional groups (mean and SD or proportion of each group).

	Physicians (*n*=20)	Physiotherapists (*n*=22)	Nurses (*n*=23)	Total sample (*n*=65)
Age (years)	44 (10)	36 (11)	34 (11)	38 (11)
Men/women	12/8	7/15	7/16	26/39
BMI (kg·m^−2^)	25 (4)	23 (3)	22 (2)	23 (3)
Working experience (years)	17 (10)	13 (11)	10 (9)	13 (10)
Proportion of HCPs with special training for LBP	85%	73%	22%	58%
Proportion of HCPs with special training for pain	90%	45%	35%	55%
Proportion of HCPs with previous experience of LBP	60%	32%	65%	52%
Proportion of HCPs with current LBP	10%	9%	30%	17%

BMI: body mass index. HCP: health care providers. LBP: low back pain.

**Table 2:** Answers for the group of experts (mean and SD, *n*=12). The severity was evaluated on a one to four Likert scale from “very mild” to “extremely severe” and the recommendations also on a one to four Likert scale from “no limitation” to “limit all PA” (level of activity) and from “full time” to “remain out of work” (level of work).

	Vignette 1	Vignette 2	Vignette 3
Severity of the symptoms	3.8 (1.0)	2.4 (0.7)	3.3 (0.5)
Severity of the pathology	2.0 (0.9)	2.0 (0.6)	2.0 (1.0)
Recommendations for the level of activity	1.9 (0.9)	1.4 (0.7)	1.5 (0.9)
Recommendations for the level of work	2.9 (0.5)	2.3 (0.8)	2.5 (1.2)

**Table 3:** Mean differences between HCPs and guidelines for the average answers of the three vignettes.

	Physicians (PY), *M* (SD) (*n*=20)	Physiotherapists (PT), *M* (SD) (*n*=22)	Nurses (NU), *M* (SD) (*n*=23)	Overall, *M* (SD) (*n*=65)	*F* (2.62)	*p*	*η* ^2^	Post hoc Scheffé
Bias_sev-sympt_	−0.9 (1.6)	0.2 (1.7)	1.0 (1.7)	0.1 (1.8)	6.95	<0.01^*∗*^	0.2 (0.03;0.3)	NU > PY
Bias_sev-patho_	0.5 (1.4)	2.2 (1.6)	1.8 (2.0)	1.5 (1.8)	6.27	<0.01^*∗*^	0.2 (0.02;0.3)	PT, NU > PY
Bias_rec-act_	1.2 (2.2)	1.9 (2.3)	4.1 (2.1)	2.5 (2.5)	10.05	<0.01^*∗*^	0.2 (0.1;0.4)	NU > PT, PY
Bias_rec-work_	0.2 (1.6)	1.9 (2.1)	2.6 (1.8)	1.6 (2.1)	9.30	<0.01^*∗*^	0.3 (0.1;0.4)	PT, NU > PY
ICC_sev_	0.57 (0.25)	0.50 (0.23)	0.46 (0.15)	0.51 (0.21)	1.45	0.24	0.04 (0;0.2)	—
ICC_rec_	0.45 (0.30)	0.42 (0.27)	0.26 (0.22)	0.37 (0.27)	3.13	0.05	0.1 (0;0.2)	—

*p* < 0.05 indicates that there was a significant difference between the groups and is labelled with a^*∗*^. A bias <0 means that the HCPs evaluated the symptoms or the pathology as less severe or recommended more activity or work than the experts. An ICC nearest of 1 means a higher adequacy with the group of experts. The column post hoc Scheffé indicates all the significant differences between two groups.

**Table 4:** Scores on the questionnaires for the three professional groups and all the subjects (mean ± SD).

Questionnaires		Physicians (PY), *M* (SD) (*n*=20)	Physiotherapists (PT), *M* (SD) (*n*=22)	Nurses (NU), *M* (SD) (*n*=23)	Overall, *M* (SD) (*n*=65)	*p*	*F*	*η* ^2^	Post hoc Scheffé
POAM-P	Avoidance	17 (9)	18 (7)	22 (6)	19 (8)	(2.62) 3.30	0.04^*∗*^	0.1 (0;0.2)	—
	Pacing	24 (10)	26 (9)	22 (8)	24 (9)	(2.62) 0.66	0.52	0.02 (0;0.1)	—
	Overdoing	26 (7)	24 (6)	24 (7)	24 (7)	(2.62) 1.03	0.37	0.03 (0;0.1)	—

TSK		31 (6)	34 (7)	40 (5)	35 (7)	(2.62) 12.99	<0.01^*∗*^	0.3 (0.1;0.4)	NU > PY, PT

HC-PAIRS		52 (7) (*n* = 14)	57 (8) (*n* = 19)	65 (9) (*n* = 22)	59 (10) (*n* = 56)	(2.51) 11.98	<0.01^*∗*^	0.3 (0.1;0.5)	NU > PY, PT

HADS	Anxiety	6 (2)	5 (2)	8 (3)	6 (2)	(2.62) 4.35	0.02^*∗*^	0.1 (0.002;0.3)	NU > PT
	Depression	2 (2)	2 (2)	4 (2)	3 (2)	(2.62) 4.20	0.02^*∗*^	0.1 (0.004;0.3)	NU > PT

IUS		45 (12)	42 (8)	52 (14)	47 (12)	(2.62) 3.71	0.03^*∗*^	0.1 (0;0.2)	NU > PY

MC-SDS		19 (5)	19 (4)	20 (4)	20 (4)	(2.62) 0.38	0.68	0.01 (0;0.1)	—

*p* value < 0.05 is labelled with a^*∗*^ which indicates that there was a significant difference between the three professional groups. The column post hoc Scheffé indicates all the significant differences between two groups.

**Table 5:** Perceived PA and objective PA for the three professional groups and all the HCPs (mean ± SD): scores on the Baecke and number of steps per day during working days and days off.

	Physicians (PY), M (SD) *n*=20	Physiotherapists (PT), M (SD) *n*=22	Nurses (NU), M (SD) *n*=23	Overall, M (SD) *n*=65	*F* (2.62)	*p*	*η* ^2^	Post hoc Scheffé
Work index	2.2 (0.4)	3.4 (0.3)	3.6 (0.6)	3.1 (0.8)	51.67	<0.01^*∗*^	0.6 (0.5;0.7)	PT, NU > PY
Sport index	3.3 (0.7)	3.5 (0.5)	2.9 (0.7)	3.2 (0.7)	5.34	0.01^*∗*^	0.1 (0.01;0.3)	PT > NU
Leisure activity index	3.3 (0.4)	3.1 (0.6)	2.9 (0.5)	3.1 (0.5)	4.23	0.02^*∗*^	0.1 (0;0.3)	PY > NU
Baecke overall	2.9 (0.3)	3.3 (0.3)	3.1 (0.3)	3.1 (0.4)	9.73	<0.01^*∗*^	0.2 (0;0.4)	PT > PY, NU
Steps/day (working day)	6,153 (1,884)	8,951 (2,885)	8,197 (2,313)	7,812 (2,699)	13.60	<0.01^*∗*^	0.3 (0.1;0.4)	PT, NU > PY

*p* value <0.05  is labelled with a^*∗*^ which indicates that there was a significant difference between the three professional groups. The column post hoc Scheffé indicates all the significant differences between two groups.

## Data Availability

The data used to support the findings of this study are available from the corresponding author upon request.
